# Genome-wide association study (GWAS) reveals genetic loci of lead (Pb) tolerance during seedling establishment in rapeseed (*Brassica napus *L.)

**DOI:** 10.1186/s12864-020-6558-4

**Published:** 2020-02-10

**Authors:** Fugui Zhang, Xin Xiao, Kun Xu, Xi Cheng, Ting Xie, Jihong Hu, Xiaoming Wu

**Affiliations:** Key Laboratory of Biology and Genetic Improvement of Oil Crops, Ministry of Agriculture and Rural Affairs, Oil Crops Research Institute of the Chinese Academy of Agricultural Sciences, Xudong 2nd Road, Wuhan, 430062 Hubei China

**Keywords:** Lead (Pb) tolerance, Phytoremediation, SNP markers, GWAS, Rapeseed

## Abstract

**Background:**

Lead (Pb) pollution in soil has become one of the major environmental threats to plant growth and human health. Safe utilization of Pb contaminated soil by phytoremediation require Pb-tolerant rapeseed (*Brassica napus* L.) accessions. However, breeding of new *B. napus* cultivars tolerance to Pb stress has been restricted by limited knowledge on molecular mechanisms involved in Pb tolerance. This work was carried out to identify genetic loci related to Pb tolerance during seedling establishment in rapeseed.

**Results:**

Pb tolerance, which was assessed by quantifying radicle length (RL) under 0 or 100 mg/L Pb stress condition, shown an extensive variation in 472 worldwide-collected rapeseed accessions. Based on the criterion of relative RL > 80%, six Pb-tolerant genotypes were selected. Four quantitative trait loci (QTLs) associated with Pb tolerance were identified by Genome-wide association study. The expression level of nine promising candidate genes, including *GSTUs*, *BCATs*, *UBP13*, *TBR* and *HIPP01*, located in these four QTL regions, were significantly higher or induced by Pb in Pb-tolerant accessions in comparison to Pb-sensitive accessions.

**Conclusion:**

To our knowledge, this is the first study on Pb-tolerant germplasms and genomic loci in *B. napus*. The findings can provide valuable genetic resources for the breeding of Pb-tolerant *B. napus* cultivars and understanding of Pb tolerance mechanism in Brassica species.

## Background

Lead (Pb) pollution in soil, from anthropogenic activities such as burning of fossil fuels, mining, discharge of untreated industrial wastes and effluents, and unreasonable disposal of lead batteries, has become a worldwide environmental issue [1, 2]. Pb in soil, is easily transferred to plant tissues, can not only influence various morphological, physiological and biochemical processes in plant, can also threats to human health through food chains [3–5]. Several alleviating techniques such as phytoremediation (including Phytostabilization and Phytoextraction) have been applied for safe utilization of Pb contaminated soil [6, 7]. Development of new cultivars tolerance to Pb toxicity will be the first step for safe utilization of Pb polluted soil by phytoremediation [8–10].

Rapeseed (*Brassica napus* L.), an ideal plant for phytoremediation, is an important source of edible vegetable oil, vegetable, animal fodder, green manure and biodiesel [11]. Breeding rapeseed cultivars with Pb-tolerant require germplasms and genetic loci related to Pb tolerance. Whereas, more and more genotypes tolerance to Pb toxicity have been selected in rice, ramie and willow populations, very few Pb-tolerant *B. napus* germplasm has been investigated [12–17]. At the vegetative and adult stage, Pb toxicity in rapeseed was evident from elevated levels of oxidative stress and subcellular damage that significantly inhibited plant growth, leaf chlorophyll contents, gas exchange parameters and photosynthetic attributes [18–21]. But at the initial growth stages (the beginning of life cycle, such as seedling establishment), serve as an important indicator in determining the toxicity effects of heavy metals (HMs) on plants, only cadmium (Cd) toxicity effect has been reported in rapeseed [22, 23].

Unlike in other plants, few data is available on molecular mechanisms involved in Pb tolerance in rapeseed. In Arabidopsis *AtACBP1* (Acyl-CoA-binding domain-containing protein), At*PSAE1* (Photosystem I reaction center subunit IV A) and several ABC (ATP-binding cassette) transporter genes (*AtATM3*, *AtPDR8*, and *AtPDR12*) have been identified as being involved in tolerance to Pb stress [24–27]. Previous research has also demonstrated that *HvCBT1* (CaM binding transporter) in barley, *AtCNGC1* (cyclic nucleotide-gated ion channel) in Arabidopsis and *NtCBP4* in tobacco, as one of the nonselective entry pathways used by Pb [28–31]. For further exploring genetic factors responding to Pb stress, Genome-wide association study (GWAS), a powerful tool to detect the genetic architecture of complex traits, has been widely used in rice, maize and grasses [12, 32–39]. GWAS has also been used to study HMs concentration, tolerance to Cd and other abiotic stress related quantitative trait loci (QTLs), but not the molecular mechanism of Pb tolerance in *B. napus* [23, 40–43].

The objectives of this study were screening elite germplasms tolerance to Pb stress at seedling establishment stage among 472 worldwide-collected rapeseed accessions and identification of QTLs and candidate genes related to Pb tolerance by GWAS for the first time in *B. napus*. The findings can provide valuable genetic resources for breeding of Pb-tolerant cultivars and understanding of the molecular mechanisms responding to Pb stress in Brassica species.

## Results

### Screening elite *B. napus* germplasms tolerance to Pb stress

**Fig. 1:**
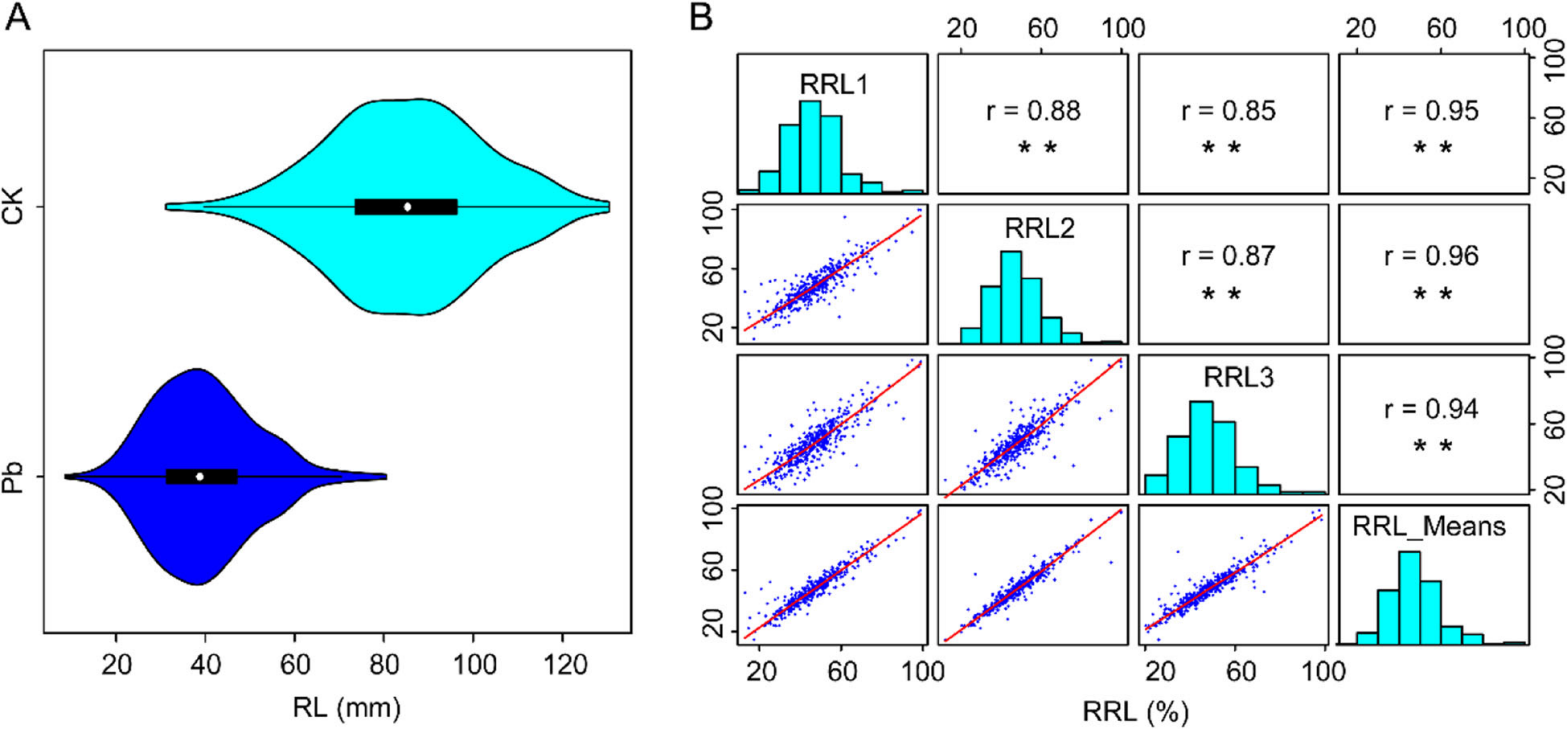
Distributions and correlation matrixes of traits. **a** Violin plot of radicle length (RL) under control (CK) and Pb stress (Pb) condition. **b** Distributions and correlation matrixes of relative radicle length (RRL). RRL1, RRL2, RRL3 represent the RRL in replication 1, 2 and 3 respectively. RRL_Means was the average value of three RRLs

To investigate the tolerance to Pb stress of different *B. napus* genotypes, the radicle lengths (RL) of 472 accessions grown under 0 or 100 mg/L Pb stress condition for seven days were compared. Although the RL varied significantly among all the accessions under both normal and Pb stress conditions (with a range from 31.15 to 130.50 mm (mm), and 8.67 to 80.60 mm, respectively), the RL of all accessions under Pb stress condition were shorter than that under normal condition (Fig. 1a, Additional file 1: Figure S1). The average of RL under normal growth condition was 85.18 ± 0.08 mm, whereas the average of RL under Pb stress condition was 39.77 ± 0.05 mm (Fig. 1a). This is consistent with previous reports [23, 44].

To eliminate the genetic variations in RLs under normal condition, the relative radicle length (RRL) was employed to evaluate the tolerance to Pb stress of *B. napus* as reported previously [23, 45]. We found that the RRL was ranged from 12.94 to 98.88, 12.17 to 99.84, 20.34 to 98.42 in three replications, respectively (Fig. 1b, Additional file 5: Table S1). And the coefficient of variation ranged from 26.37 to 28.57% in three replications (Additional file 5: Table S1). These results indicate that this *B. napus* population exhibited a broad variation of Pb tolerance.

To select stable Pb-tolerant genotypes for potentially used in phytoremediation or new cultivar breeding, we performed correlation analyses, and found that the RRLs of three replications were significantly correlated with each other with a correlation coefficient value over 0.85 (Fig. 1b). Based on the values of RRLs of all the accessions, six Pb-tolerant genotypes (RRL > 80%) were selected (Additional file 6: Table S2).

### Detection of QTLs associated with Pb tolerance

To select a most suitable model for GWAS analysis of Pb tolerance in the population, the native, population structure (Q), principal component analysis (P), kinship (K), Q + K and P + K models were tested. As shown in quantile-quantile plots (Q-Q) plot, the distribution of observed −log10(*p*) from Q + K model provided the best fit with the expected distribution (Additional file 2: Figure S2). Therefore, to decrease the rate of false-positive, Q + K model was chosen for subsequent analysis.

**Fig. 2:**
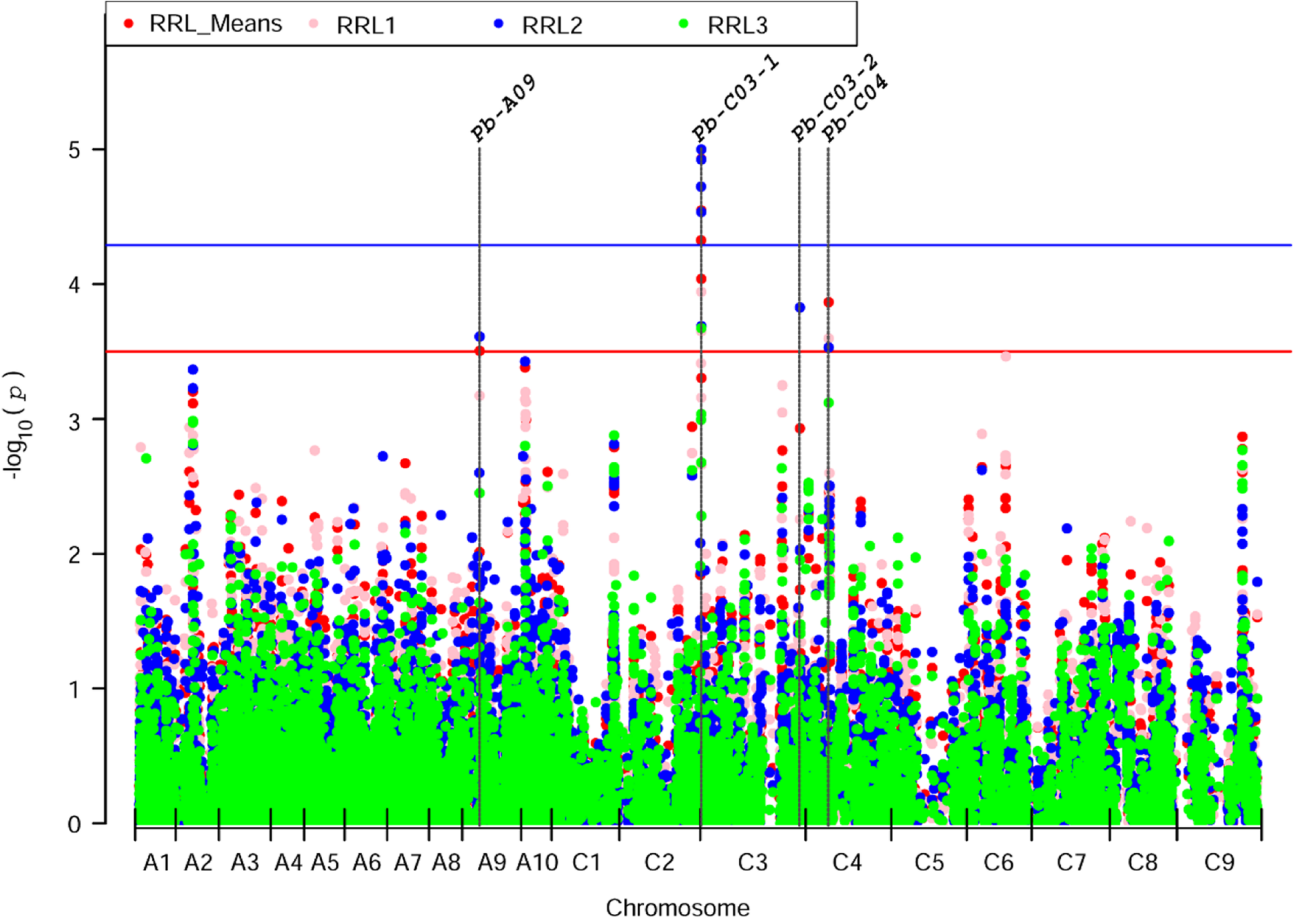
Manhattan plots of association analysis for RRLs using Q + K model. The red, pink, blue and green dots represent the association signals for RRL_Means (average value of three RRLs), RRL1 (RRL in replication 1), RRL2 (RRL in replication 2) and RRL3 (RRL in replication 3), respectively. The blue and red horizontal lines indicate the significantly associated threshold (−log10(1/19,945) = 4.3) and moderately associated threshold (−log10(*p*) between 3.5–4.3), respectively

**Fig. 3:**
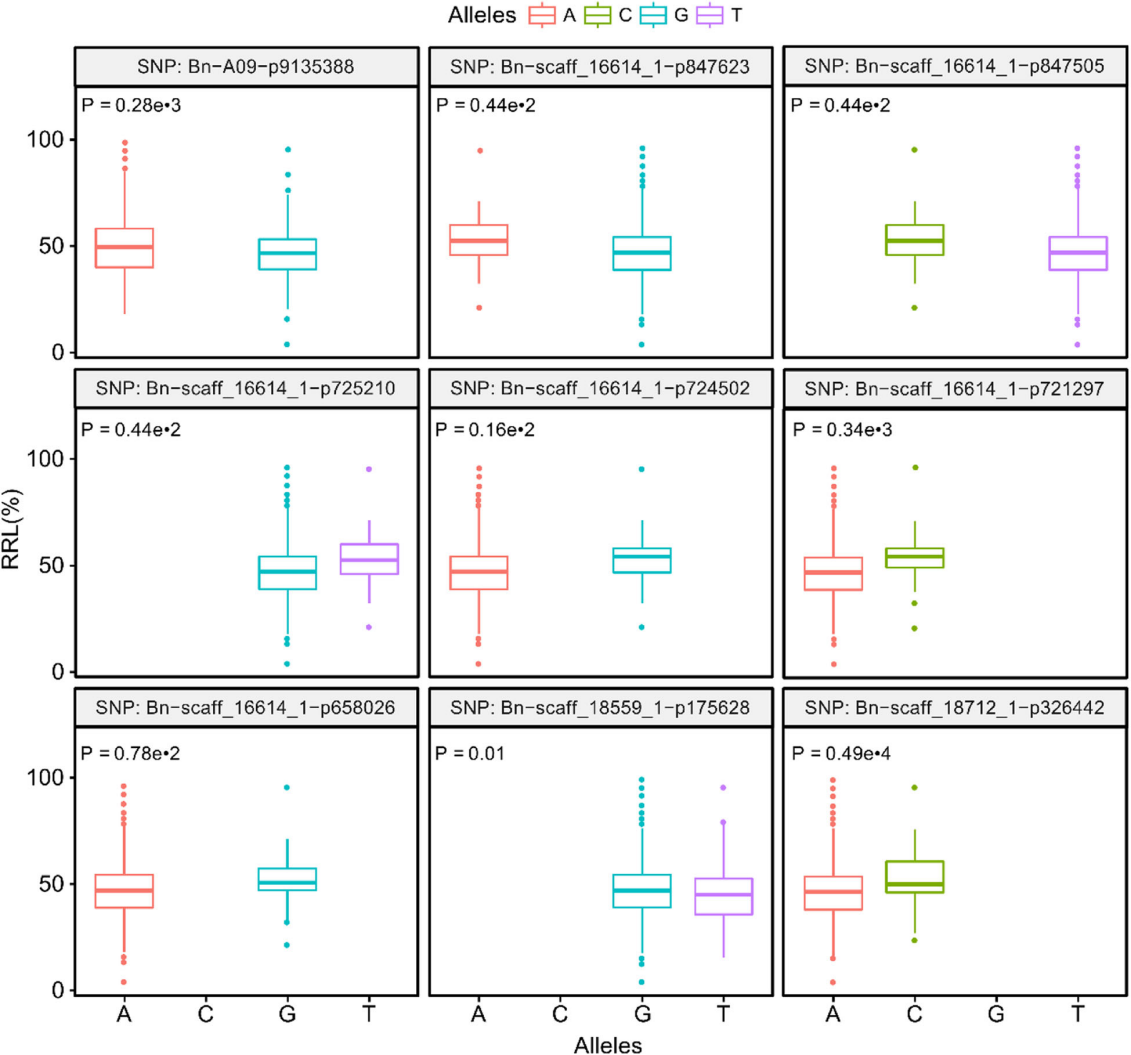
Allele effects of associated SNPs. Red, green, blue and purple boxes indicated the A, C, G and T alleles, respectively. The “*P*” presents significant different level of RRL between alleles by *t-* test

Six significantly associated single nucleotide polymorphisms (SNPs) (−log10(*p*) > 4.3) and three moderately associated SNPs (3.5 < −log10(*p*) < 4.3) located on chromosome A09, C03 and C04 were detected (Fig. 2). Almost all of them (except for Bn-scaff_16614_1-p658026 and Bn-scaff_18559_1-p175628) were identified in more than two replications, and four out of the nine SNPs were detected in all replications (Table 1). In addition, the significant difference of RRLs between alleles in all nine SNPs were confirmed by *t*-test (Fig. 3).

**Table 1:** Genome-wide association signals of Pb tolerance

Marker informations				Association analysis		
Markers	Chromosomes	Positions	Alleles	-Log(*p*)	MarkerR^2^	Traits
Bn-A09-p9135388	A09	8,316,886	A/G	3.73	3.81	RRL2,RRL_Means
Bn-scaff_16614_1-p847623	C03	1,241,778	A/G	4.84	5.34	RRL1,RRL2,RRL3,RRL_Means
Bn-scaff_16614_1-p847505	C03	1,241,796	T/C	4.84	5.34	RRL1,RRL2,RRL3,RRL_Means
Bn-scaff_16614_1-p725210	C03	1,377,666	T/G	4.56	5.07	RRL2,RRL_Means
Bn-scaff_16614_1-p724502	C03	1,378,275	A/G	4.95	5.46	RRL1,RRL2,RRL3,RRL_Means
Bn-scaff_16614_1-p721297	C03	1,381,475	A/C	5.1	5.61	RRL1,RRL2,RRL3,RRL_Means
Bn-scaff_16614_1-p658026	C03	1,446,328	A/G	3.8	4.21	RRL2
Bn-scaff_18559_1-p175628	C03	58,079,114	T/G	4.33	4.43	RRL2
Bn-scaff_18712_1-p326442	C04	14,028,410	A/C	4.25	4.31	RRL1,RRL2,RRL3,RRL_Means

RRL1, RRL2, RRL3 represent the relative RRL in replication 1, 2 and 3 respectively. RRL_Means was the average value of three RRLs

**Fig. 4:**
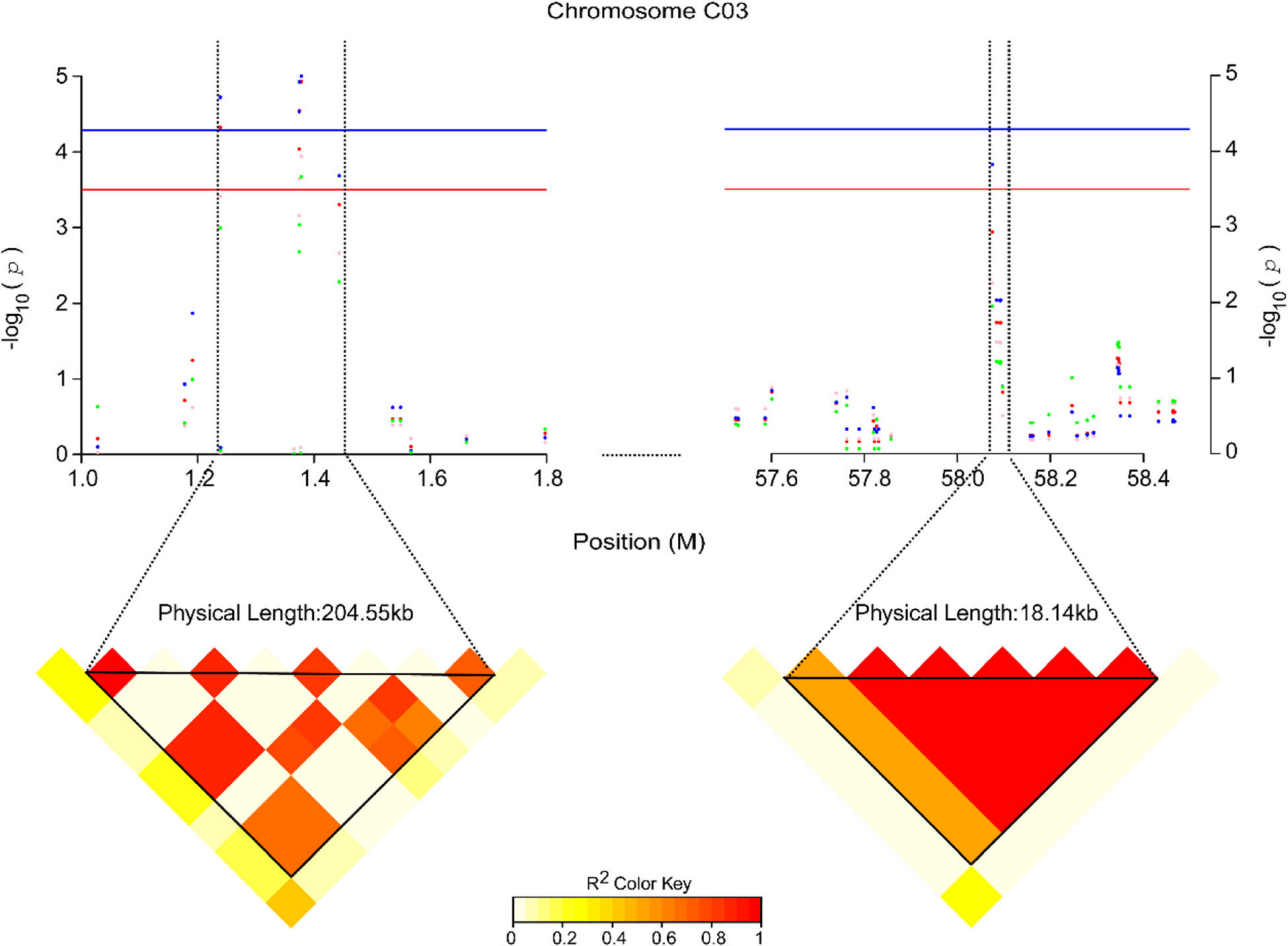
Association mapping for RRL on chromosome C03. Plots show the SNPs in the QTL *Pb-C03–1* (top left of Fig. 4, from 1,241,778 to 1,446,328 bp on chromosome C03) and *Pb-C03–2* (top right of Fig. 4, from 58,079,114 to 58,097,249 bp on chromosome C03) regions associated with RRL. The red, pink, blue and green dots represent the association signals for RRL_Means (average value of three RRLs), RRL1 (RRL in replication 1), RRL2 (RRL in replication 2) and RRL3 (RRL in replication 3), respectively. The blue and red horizontal line indicate the threshold of significantly associated SNPs at −log10 (1/19,945) = 4.3 and threshold of moderately associated SNPs at 3.5 ≤ −log10 (*p*) ≤ 4.3, respectively as in Fig. 2. The heat maps span the linkage disquilibrium (LD) region with the most strongly associated SNPs (*r*^*2*^ > 0.4)

Further studies with linkage disequilibrium (LD) analyses indicated that these nine associated signals were located in four QTLs. QTL *Pb-C03–1* (204.55 kb, position from 1,241,778 bp to 1,446,328 bp on chromosome C03) contained six SNPs, with a peak SNP Bn-scaff_16614_1-p721297 which gave a 5.61% contribution to the phenotypic variance (Fig. 4, Table 1). Whereas, QTL *Pb-A09* (265.76 kb, position from 8,148,958 bp to 8,414,720 bp on chromosome A09, Additional file 3: Figure S3a), QTL *Pb-C03–2* (18.14 kb, position from 58,079,114 bp to 58,097,249 bp on chromosome C03, Fig. 4) and QTL *Pb-CO4* (186.37 kb, position from 14,028,410 bp to 14,214,776 bp on chromosome C04, Additional file 3: Figure S3b) all contained only one associated SNP, and respectively gave a 3.81, 4.43 and 4.31% contribution to the phenotypic variance (Table 1).

### Identification of candidate genes related to Pb tolerance

For the identification of candidate genes related to Pb tolerance, all the 115 genes located in the QTL regions (29, 41, 24 and 21 genes in QTL regions *Pb-A09*, *Pb-C03–1*, *Pb-C03–2* and *Pb-C04*, respectively) were annotated by nucleic acid basic local alignment search tool (BLASTN) with *A. thaliana* genome and Kyoto Encyclopedia of Genes and Genomes (KEGG) databases. The top 20 enriched metabolic pathways were shown in Additional file 4: Figure S4 and Additional file 7: Table S3. Based on the criterion qvalue ≤0.05, three genes, *BnaA09g14510D*, *BnaA09g14520D* and *BnaA09g14540D*, enriched in glutathione metabolism pathway, and three genes, *BnaC03g68440D*, *BnaC03g68450D*, and *BnaC03g68460D*, enriched in the biosynthesis pathway of pantothenate and CoA, as well as in the biosynthesis degradation pathways of valine, leucine and isoleucine were selected for further analyses (Additional file 7: Table S3). The other three candidate genes, *BnaC03g02630D*, *BnaC03g02690D* and *BnaC04g16200D*, which were homologous with *AtUBP13* (ubiquitin-specific protease 13), *AtTBR* (Trichome birefringence) and *AtHIPP01* (heavy metal-associated isoprenylated plant protein) respectively, were also selected for further analyses. All these nine candidate genes may contribute to Pb tolerance in *B. napus* by regulating glutathione metabolism, cell wall development, ubiquitination and amino acid metabolism, respectively (Table 2).

**Table 2:** A list of the most promising candidate genes for Pb tolerance in rapeseed

QTLs	Candidate Genes	Locations	Distance to associated SNPs (kb)	*A. thaliana* orthologs	Annotations
*Pb-A09*	*BnaA09g14510D*	strand - (chrA09:8337575..8338088)	20.69	*AT1G59700*	*AtGSTU16*, Glutathione S-transferase
	*BnaA09g14520D*	strand - (chrA09:8345181..8345671)	28.3	*AT1G59700*	*AtGSTU16*, Glutathione S-transferase
	*BnaA09g14540D*	strand - (chrA09:8372673..8373944)	55.79	*AT1G59670*	*AtGSTU15*, Glutathione S-transferase.
*Pb-C03–1*	*BnaC03g02630D*	strand - (chrC03:1250190..1259000)	17.204	*AT3G11910*	*AtUBP13*,ubiquitin-specific protease 13
	*BnaC03g02690D*	strand + (chrC03:1282603..1284862)	43.066	*AT5G06700*	*AtTBR*, Protein trichome birefringence
*Pb-C03–2*	*BnaC03g68440D*	strand - (chrC03:58120344..58122336)	43.22	*AT1G50110*	*AtBCAT6*, Branched-chain-amino-acid aminotransferase 6,
	*BnaC03g68450D*	strand - (chrC03:58123734..58126490)	47.38	*AT1G50090*	*AtBCAT7*, Putative branched-chain-amino-acid aminotransferase 7.
	*BnaC03g68460D*	strand - (chrC03:58136020..58137057)	57.94	–	–
*Pb-C04*	*BnaC04g16200D*	strand + (chrC04:14207994..14209539)	179.58	*AT2G28090*	AtHIPP01, Heavy metal-associated isoprenylated plant protein 1

### Exploring the expression level of candidate genes

**Fig. 5:**
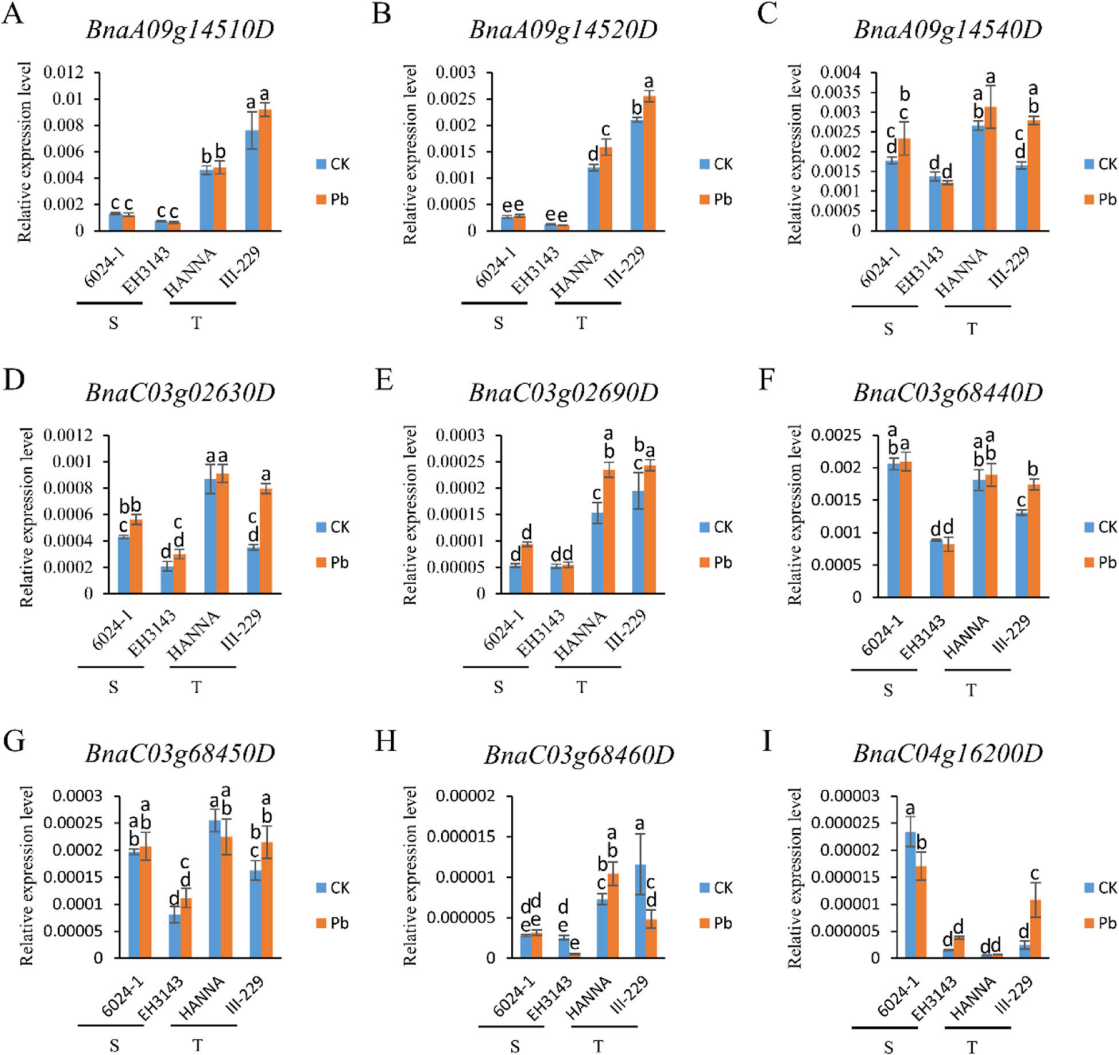
The relative expression level of candidate genes for Pb tolerance. The “a, b, c, d and e” present significant difference at 5% level by tukey test

To investigate the expression levels of these candidate genes under both normal and Pb stress conditions in both Pb-tolerant and Pb-sensitive accessions, we performed quantitative real time polymerase chain reaction (qRT-PCR) assay. We observed that the expression level of *BnaA09g14520D*, *BnaA09g14520D* and *BnaA09g14540D* located in QTL *Pb-A09*, and *BnaC03g02630D* and *BnaC03g02690D* located in QTL *Pb-C03–1*, were extremely higher in Pb-tolerant genotypes than in Pb-sensitive genotypes (Fig. 5a, b, c, d, e). *BnaA09g14520D* and *BnaC03g02690D* were significantly induced by Pb stress only in two Pb-tolerant accessions (Fig. 5b, e). *BnaA09g14540D* and *BnaC03g02630D* were significantly up-regulated in a Pb-tolerant accession III-229 and only slightly up-regulated in the other accessions under Pb stress (Fig. 5c, d).

*BnaC03g68440D*, *BnaC03g68450D* and *BnaC03g68460D* located in QTL *Pb-C03–2* were enriched in the same pathways. We found that *BnaC03g68440D* and *BnaC03g68450D* were significantly induced by Pb stress in III-229 (Fig. 5f, g), and the expression levels of *BnaC03g68440D* and *BnaC03g68450D* in Pb-sensitive genotype EH3143 were extensively lower in comparison to Pb-tolerant genotypes (Fig. 5f, g). Similarly, a higher expression level of *BnaC03g68460D* was also observed in the two Pb-tolerant genotypes than in two Pb-sensitive genotypes (Fig. 5h). Under Pb stress condition, *BnaC04g16200D*, located in QTL *Pb-C04*, was remarkably up-regulated in Pb-tolerant genotype III-229 and down-regulated in Pb-sensitive genotype 6024–1 (Fig. 5i).

## Discussions

### Pb-tolerant accessions provide valuable resources for phytoremediation

Pb, as known to be a non-essential HMs, causes a series of severe phyto-toxicities including growth inhibition, declines in photosynthesis, respiration and mineral nutrition, and even death. Especially in the initial stages, seed germination and seedling establishment were extremely inhibited by high concentration of Pb stress [22, 46]. In this study we also found that the RL of *B. napus* was seriously short under Pb stress in comparison to under normal condition (Fig. 1a, Additional file 1: Figure S1) during seedling establishment. This phenomenon is principally because radicle is the first tissue of plants exposed to HMs [23, 47].

Pb tolerance, represent the ability of plants to adapt to and cope with Pb stress, was commonly evaluated by relative growth indexes under both normal and Pb stress conditions [45]. Considering the severe inhibition of Pb stress on radicle elongation, the RRL has been employed to evaluate the tolerance of *B. napus* to Pb stress. Extensive phenotypic variation for Pb tolerance in *B. napus* population (Fig. 1b, Additional file 5: Table S1), as well as HMs tolerance in many other plant species, has been observed [47–49]. Six Pd-tolerant genotypes (Additional file 6: Table S2) selected from the population can provide valuable plant resources which is usable for the breeding of Pb-tolerant *B. napus* cultivars [6, 9].

### Specific QTLs for Pb tolerance were identified in *B. napus*

To detect Pb tolerance related QTLs by GWAS in *B. napus*, the Q + K model which was also used in seed weight and seed quality, branch angle and flowering time studies, was utilized in this study [50–52]. Nine associated signals located in four QTLs were obtained (Fig. 2, Table 1). To determinate whether these four QTLs is specific for Pb tolerance in *B. napus*, comparison analysis was conducted. We found that no QTL was overlapped with previous reported Cd responsive QTLs in *B. napus* [23, 40], although several protein such as AtHMA2 (Heavy Metal ATPases) and AtPDR8 can transport both Cd and Pb in plant [25, 53]. This might be caused by the different populations used for GWAS and the large difference of genetic factors between Pb and Cd stress responses [9, 54]. Thus, the four QTLs might be specific genetic factors for tolerance to Pb stress in *B. napus.*

### Higher expression of GSTs contributes to Pb-tolerant

Glutathione S-transferases (GSTs) contributed to HMs tolerance mainly by playing important roles in the cellular antioxidant defense mechanisms and serving as non-enzymatic carriers for intracellular transport [55, 56]. We identified three *GSTs* genes, *BnaA09g14510D*, *BnaA09g14520D*, and *BnaA09g14540D*, in QTL *Pb-A09* (Table 2). qRT-PCR assays demonstrated that the expression levels of these three genes were extremely higher in Pb-tolerant genotypes than in Pb-sensitive genotypes (Fig. 5a, b and c). Furthermore, an induced expression of *BnaA09g14520D* and *BnaA09g14540D* by Pb exposure in Pb-tolerant accessions were also observed as reported previously [55]. Therefore, increasing the activity of GSTs might be an efficient way to develop hyper-tolerant *B. napus* for phytoremediation [56, 57].

### Ubiquitination and de-ubiquitination co-regulate Pb tolerance

In QTL *Pb-A03–1*, *BnaC03g02630D* is homologous with *AtUBP13* (Table 2). *AtUBP13*, similar to *AtUBP16*, *AtUBP6*, *ZmUBP15*, *ZmUBP16* and *ZmUBP19*, which can increase plant tolerance to HMs stress, all belong to the de-ubiquitinating enzymes family [53, 58–60]. In our study, the expression level of *BnaC03g02630D* was significantly higher in Pb-tolerant accessions than in Pb-sensitive accessions (Fig. 5d). Whereas, *NtUBC1 and* GmARI1, which can modify protein by ubiquitin, can also enhance HMs tolerance in plants [61, 62]. We infer that both modification of protein by ubiquitin and de-ubiquitin can alleviate HMs toxicity, in which the target proteins may be the critical factor for HMs tolerance in plant. Further studies will be conducted to investigate the targets of *BnaC03g02630D* to increase the tolerance of *B. napus* to HMs stress.

### TBR protein was associated with Pb tolerance by regulating cell wall development

Trichome birefringence (TBR) contributes to the synthesis and deposition of secondary wall cellulose, and helps to maintain the esterification of pectin [63, 64]. It has been demonstrated that increasing cell wall capacity for the compartmentalization of Pb is a major approach for plant cell to protect protoplasts from Pb toxicity [9, 65–67]. In this study, *BnaC03g02690D*, a homology of *TBR* (*AT5G06700*) gene, was also identified in QTL *Pb-A03–1* (Table 2). The expression level of *BnaC03g02690D* was significantly higher and induced by Pb in Pb-tolerant accessions than in Pb-sensitive accessions (Fig. 5e). Therefore, the TBR protein encoded by *BnaC03g02690D* contribute to Pb detoxification by increasing cell wall capacity through the compartmentalization of Pb in *B. napus*.

### BCAA metabolism regulation can mediate Pb tolerance

Branched-chain-amino-acid aminotransferase (BCAT), which catalyzes both the last anabolic step and the first catabolic step of branched-chain-amino-acids (BCAAs, including valine, leucine and isoleucine) metabolism, can mediate HMs tolerance in plant [68–71]. In QTL *Pb-C03–2*, *BnaC03g68440D*, *BnaC03g68450D*, and *BnaC03g68460D*, enriched in the biosynthesis pathway of pantothenate and CoA, as well as in the biosynthesis degradation pathways of valine, leucine and isoleucine (Additional file 7: Table S3). *BnaC03g68440D* and *BnaC03g68450D*, which encoded a BCAT, were highly induced by Pb in Pb-tolerant accession III-229 (Fig. 5f, g). The expression level of *BnaC03g68460D* was higher in Pb tolerance genotypes than in Pb-sensitive genotypes (Fig. 5h). ALL these results suggest that these three genes, detected in QTL *Pb-C03–2*, contribute to Pb tolerance of *B. napus* by regulating BCAAs metabolism.

### *BnaHIPP01* might contribute to detoxification of Pb stress

It is well known that HIPPs, containing HM–binding domain (HMA, pfam00403.6), have important functions in plant responses to both biotic and abiotic stresses [72, 73]. In *Arabidopsis*, the *AtHIPP20*, *AtHIPP22*, *AtHIPP26* and *AtHIPP27* genes were involved in Cd detoxification [74, 75]. We found that *BnaC04g16200D*, the homolog of *AtHIPP01*, was significantly up-regulated in Pb-tolerant genotype III-229 and down-regulated in Pb-sensitive genotype 6024–1 under Pb stress (Fig. 5i). These findings suggest that, *BnaC04g16200D* might contribute to the detoxification of Pb stress, as did *BnHIPP27* to Cd stress in *B. napus* [23].

## Conclusions

To our knowledge, this is the first study on Pb-tolerant germplasms and genomic loci in *B. napus*. We found that Pb tolerance shown an extensive variation in 472 worldwide-collected rapeseed accessions. Based on the criterion of relative RL > 80%, six Pb-tolerant genotypes were selected. Four QTLs associated with Pb tolerance were identified by GWAS. Nine promising candidate genes, including *GSTUs*, *BCATs*, *UBP13*, *TBR* and *HIPP01*, located in these four QTL regions were selected. The expression level of these nine genes were significantly higher or induced by Pb in Pb-tolerant accessions in comparison to Pb-sensitive accessions. These findings can provide valuable genetic resources for the breeding of Pb-tolerant *B. napus* cultivar and understanding of Pb tolerance mechanism in Brassica species.

## Methods

### Pb tolerance evaluation of 472 *B. napus* accessions

A total of 472 representative *B. napus* accessions (266 accessions originated from Asia, 128 from Western Europe, 20 from Oceania, 26 from North America and 32 from Eastern Europe, Additional file 8: Table S4) used for association analysis were collected from the National Mid-term Gene Bank for Oil Crops in Wuhan, China, as described previously [47]. In order to find out the optimal method for Pb tolerance evaluation at seedling establishment stage in rapeseed, we first performed a trial. The radicle length (RL) of six randomly-selected rapeseeds under 100 mg/L Pb stress was showed wider variation than under other Pb stress groups (50 mg/L and 200 mg/L). Thus, 100 mg/L was selected as an optimal concentration for screening Pb-tolerant rapeseed accessions at seedling establishment stage.

For decreasing experiment error, the 472 germplasms were split into several sets (about 40 accessions per set). In each set, seeds of these accessions were sterilized with 70% ethyl alcohol for 5 min, then rinsed at least three times with distilled water. Per treatment, fifty seeds of each accession were sown in petri dishes with four layers of filter paper soaked in 20 ml deionized water supplemented with 0 or 100 mg/L Pb. Seeds were kept in dark for two days at 23 °C with a relative humidity of 60–70%, then in 16 h light/8 h dark photoperiod with a light intensity of 300 μmol m^− 2^ s^− 1^ for another five days. All the treatments were replicated three times in a growth chamber (MLR-352H-PC, Panasonic) with less temperature fluctuation (±0.3 °C).

The RL of seven-day-old seedlings were measured with a ruler. The RRL were calculated based on the RL under control (RL_CK) and Pb stress (RL_Pb) condition with the formula RRL (%) = (RL_Pb / RL_CK) × 100. The RRL_Means of each accession were calculated by RRL values of three replications. Accessions with higher RRL were genotypes more tolerant to Pb stress. The distributions of RL and RRL were plotted using ggplot package in R software [76].

### Genome-wide association study

Genotypic data of Single-nucleotide polymorphism (SNP) had been implemented with 60 K Brassica Infinium® SNP array in previous reports [50, 77]. In which genotypic data was controlled by 12 doubled haploid (DH) lines to avoid false rate of heterozygous calls and paralogues or homeologues confusing the genotype analysis. SNP markers that appeared heterozygous within any of these DH lines were excluded from our analysis. SNPs with AA or BB frequency equal to zero, call frequency < 0.8 or minor allele frequency < 0.05 were excluded [50]. The probe sequences of remaining SNPs were used to perform a BlastN search against *B. napus* genome sequences [78]. Only the top blast-hits with an E value cut-off of 1E-15 against the *B. napus* genome sequences were considered. Furthermore, blast matches to multiple loci with the same E-value were excluded. At finally, a total of 19,945 high-quality SNP markers were used for following analysis.

The principal component analysis (P), population structure (Q) and kinship (K) matrix of the GWAS population were estimated by GCTA tool, STRUCTURE 2.3.4, and TASSEL 4.0, respectively as described in our previous studies [50, 77]. TASSEL 4.0 was used to perform GWAS analysis with the native, Q, P, K, Q + K and P + K models [79]. The native, Q and P model were performed using a general linear model with the following equation: y = Xα + e. The K, Q + K and P + K model were performed using a mixed linear model with the equation y = Xα + Kμ + e. In these equations, y represented phenotype, X represented genotype, α was a vector containing fixed effects, K was the relative kinship matrix, μ was a vector of random additive genetic effects, and e was the unobserved vector of random residual.

Q-Q plots and Manhattan plots were constructed using qqman package in R software [80]. To reduce the rate of false-positive, the best fitted mixed linear model was selected for following analysis based on the results of Q-Q plots. Significant association threshold was estimated as −log10(*p*) = 4.3 (*p* = 1/N, where N = the number of SNPs used). Besides, to avoid ignoring the effects of minor loci, SNPs passing the threshold of 3.5 were also selected for subsequent analysis as described previously [52]. The differentiation analysis of RRLs between associated SNPs’ alleles was also performed using ggpubr package in R software to control false positive.

### Identification of candidate genes

The associated QTLs were defined across regions of SNPs with LD value (r^2^) > 0.4 between the peak SNPs and surrounding SNPs using the LDheatmap package in R software. To further identify the candidate genes for Pb tolerance, the flanking SNPs outside and adjacent to the blocks were considered as the candidate regions’ boundaries. The local manhattan plots of QTLs were drawn using qqman package in R software. Genes located in the candidate regions were obtained from the reference genome of the *B. napus* “Darmor-Bzh” (http://www.genoscope.cns.fr/brassicanapus/) [78]. Pathway enrichment analysis was also employed for gene annotation in which genes in the candidate regions were blast to the KEGG database (http://www.genome.jp/) [81]. Rich factor was used to represents enrichment intensiveness, which means the ratio of the numbers of candidate genes and whole genome genes have been annotated in specific pathways. Qvalue, calculated by BH multiple test, was used for determining the threshold of *P* value. Pathways with qvalue less than 0.05 are significantly enriched. For identifying more candidate genes, all genes located in the candidate regions were also annotated by performing BLASTN in *A. thaliana* genomic database (https://www.arabidopsis.org/) [82].

### Gene expression level analysis

The expression level of candidate genes in two Pb-tolerant (HANNA and III-229) and two Pb-sensitive (6024–1 and EH3143) genotypes were evaluated by quantitative real time PCR (qRT-PCR). Total RNA was extracted from seven-day-old radicles grown under normal (0 mg/L) or Pb stress (100 mg/L) condition using TransZol kit (Trans Gene Biotech). A total amount of 500 ng RNA was used to synthesize first strand cDNA using HiScript® II Q Select RT SuperMix for qPCR (+gDNA wiper) kit (Vazyme Biotech). The gene copy specific primers of candidate genes were designed using Primer Premier 5 (Additional file 9: Table S5). The qRT-PCR assay was carried out using LightCycler® 480 SYBR Green I Master kit (Roche Life Science) in LightCycler® 480 qPCR machine (Roche Life Science) according to the manufacturer instructions. Data were collected from three technical replicates. The relative expression level was normalized by *BnACTIN7* using a △CT method (Li et al., 2017). The tukey test was employed for differentiation analysis on relative gene expression level between accessions and treatments.

## Supplementary information

**Additional file 1: Figure S1.** Histogram of radicle length (RL) under control (CK) and Pb stress (Pb) condition.

**Additional file 2: Figure S2.** The quantile–quantile plot (QQ-plot) of different models for RRL.

**Additional file 3: Figure S3.** Association mapping for RRL on chromosome A09 and C04**.** (A) Association mapping for RRL in the QTL *Pb-A09* (from 8,148,958 to 8,414,720 bp on chromosome A09). (B) Association mapping for RRL in the QTL *Pb-C04* (from 14,028,410 to 14,214,776 bp on chromosome C04) associated with RRL. The red, pink, blue and green plots represent the association signals for RRL_Means (average value of three RRLs), RRL1 (RRL in replication 1), RRL2 (RRL in replication 2) and RRL3 (RRL in replication 3), respectively. The blue and red horizontal line indicate the threshold of significantly associated SNPs at −log10 (1/19,945) = 4.3 and threshold of moderately associated SNPs at 3.5 ≤ −log10 (*p*) ≤ 4.3, respectively as in Fig. 2. The heat maps span the linkage disquilibrium (LD) region with the most strongly associated SNPs (*r*^*2*^ > 0.4).

**Additional file 4: Figure S4.** The top 20 enriched pathways of genes in the associated regions.

**Additional file 5: Table S1.** The phenotypic variation for RRLs in this natural rapeseed population. SE, Standard Error; CV, Coefficient of variation; RRL1, RRL2, RRL3 represent the relative RRL in replication 1, 2 and 3 respectively. RRL_Means was the average value of three RRLs; ** indicate significant correlation at the 1% level.

**Additional file 6: Table S2.** The details Pb-tolerant genotypes screeneded from 472 global-coolected rapeseeds. RL_CK and RL_Pb represent the radicle length (RL) under control and Pb stress (100 mg/L), respectively; RRL represent the relative radicle length.

**Additional file 7: Table S3.** The information for top 20 enriched pathways for genes in the associated regions.

**Additional file 8: Table S4.** List of 472 rapeseed accessions used for association study.

**Additional file 9: Table S5.** The primer sequences of qRT – PCR.

## Data Availability

All data associated with this study are available under the additional files data sets.

## Abbreviations

ABC: ATP-binding cassette
*ACBP*: Acyl-CoA-binding domain-containing protein
BCAAs: Branched-chain-amino-acids
BCAT: Branched-chain-amino-acid aminotransferase
BLASTN: Nucleic acid basic local alignment search tool
*CBT*: CaM binding transporter
*CNGC*: Cyclic nucleotide-gated ion channel
DH: Doubled haploid
GSTs: Glutathione S-transferases
GWAS: Genome-wide association study
*HIPP*: Heavy metal-associated isoprenylated plant protein
HMA: Heavy Metal ATPases
HMs: Heavy metals
K: Kinship
KEGG: Kyoto Encyclopedia of Genes and Genomes
LD: Linkage disequilibrium
mm: Millimeter
P: Principal component analysis
Pb: Lead
*PSAE1*: Photosystem I reaction center subunit IV A
Q: Population structure
qRT-PCR: Quantitative real time polymerase chain reaction
QTL: Quantitative trait locus
RL: Radicle lengths
RRL: Relative radicle lengths
SNP: Single nucleotide polymorphism
*TBR*: Trichome birefringence
*UBP*: Ubiquitin-specific protease
