# Supplementary figures and images for: Genome-wide association study (GWAS) reveals genetic loci of lead (Pb) tolerance during seedling establishment in rapeseed (Brassica napus L.)
Source: BMC Genomics. 2020 Feb 10;21:139. doi: 10.1186/s12864-020-6558-4 (PMC7011513; doi:10.1186/s12864-020-6558-4)

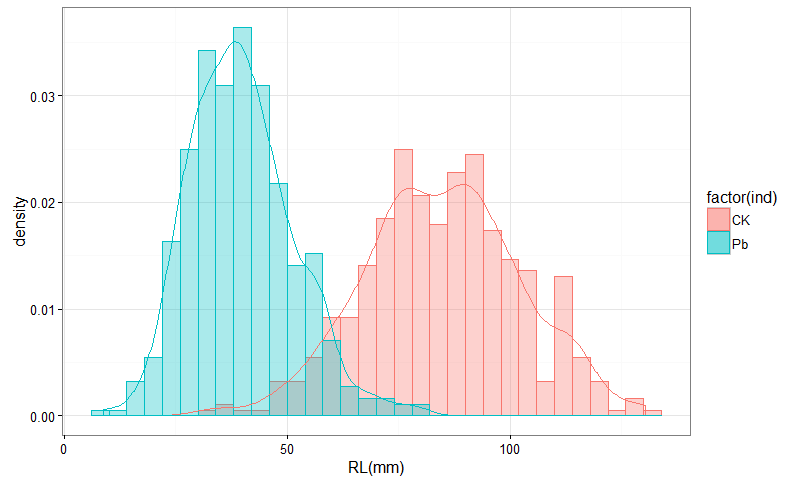

Supplement: Supplementary file 1 — Additional file 1: Figure S1. Histogram of radicle length (RL) under control (CK) and Pb stress (Pb) condition. [file 12864_2020_6558_MOESM1_ESM.tiff]

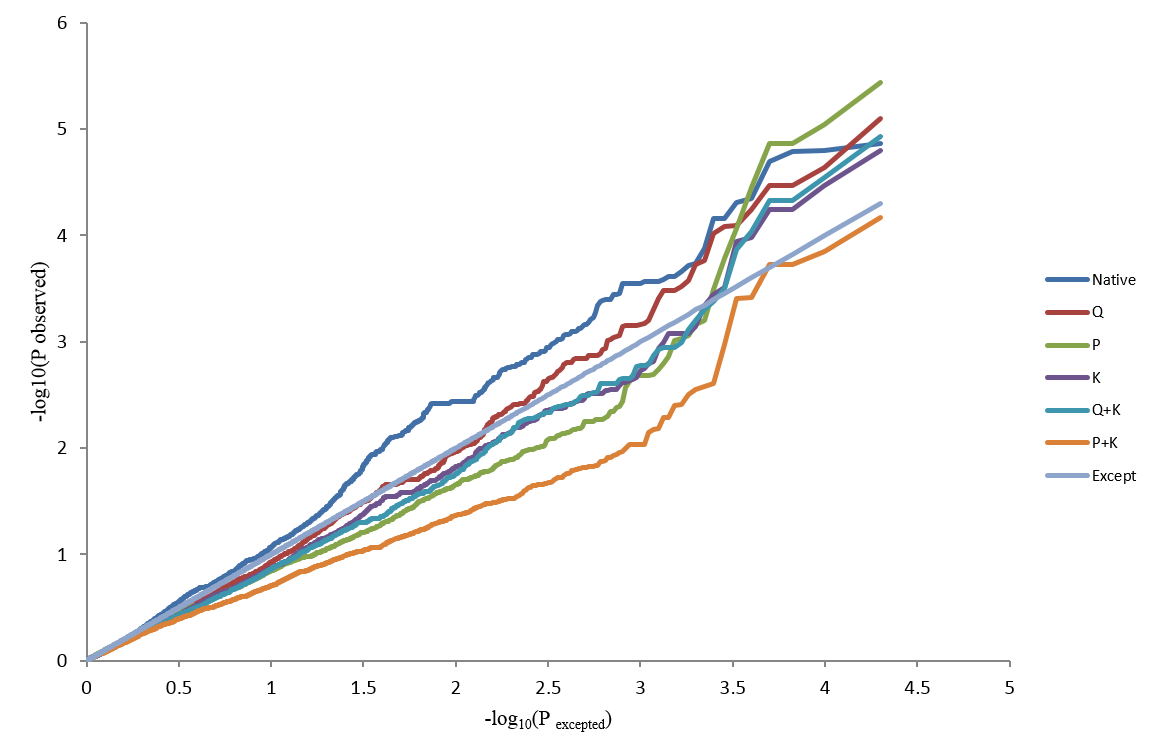

Supplement: Supplementary file 2 — Additional file 2: Figure S2. The quantile–quantile plot (QQ-plot) of different models for RRL. [file 12864_2020_6558_MOESM2_ESM.tif]

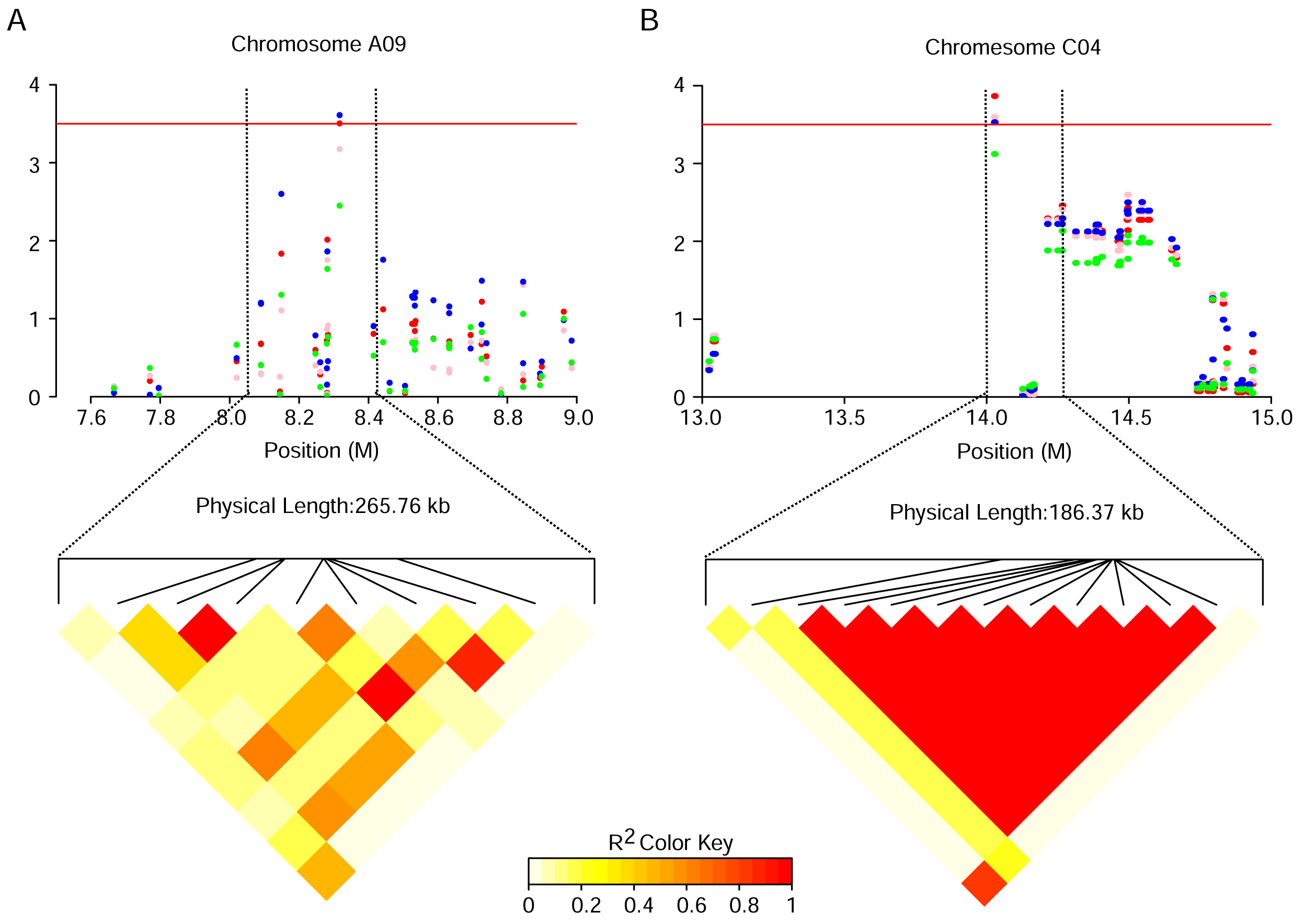

Supplement: Supplementary file 3 — Additional file 3: Figure S3. Association mapping for RRL on chromosome A09 and C04. (A) Association mapping for RRL in the QTL Pb-A09 (from 8,148,958 to 8,414,720 bp on chromosome A09). (B) Association mapping for RRL in the QTL Pb-C04 (from 14,028,410 to 14,214,776 bp on chromosome C04) associated with RRL. The red, pink, blue and green plots represent the association signals for RRL_Means (average value of three RRLs), RRL1 (RRL in replication 1), RRL2 (RRL in replication 2) and RRL3 (RRL in replication 3), respectively. The blue and red horizontal line indicate the threshold of significantly associated SNPs at −log10 (1/19,945) = 4.3 and threshold of moderately associated SNPs at 3.5 ≤ −log10 (p) ≤ 4.3, respectively as in Fig. 2. The heat maps span the linkage disquilibrium (LD) region with the most strongly associated SNPs (r2 > 0.4). [file 12864_2020_6558_MOESM3_ESM.tif]

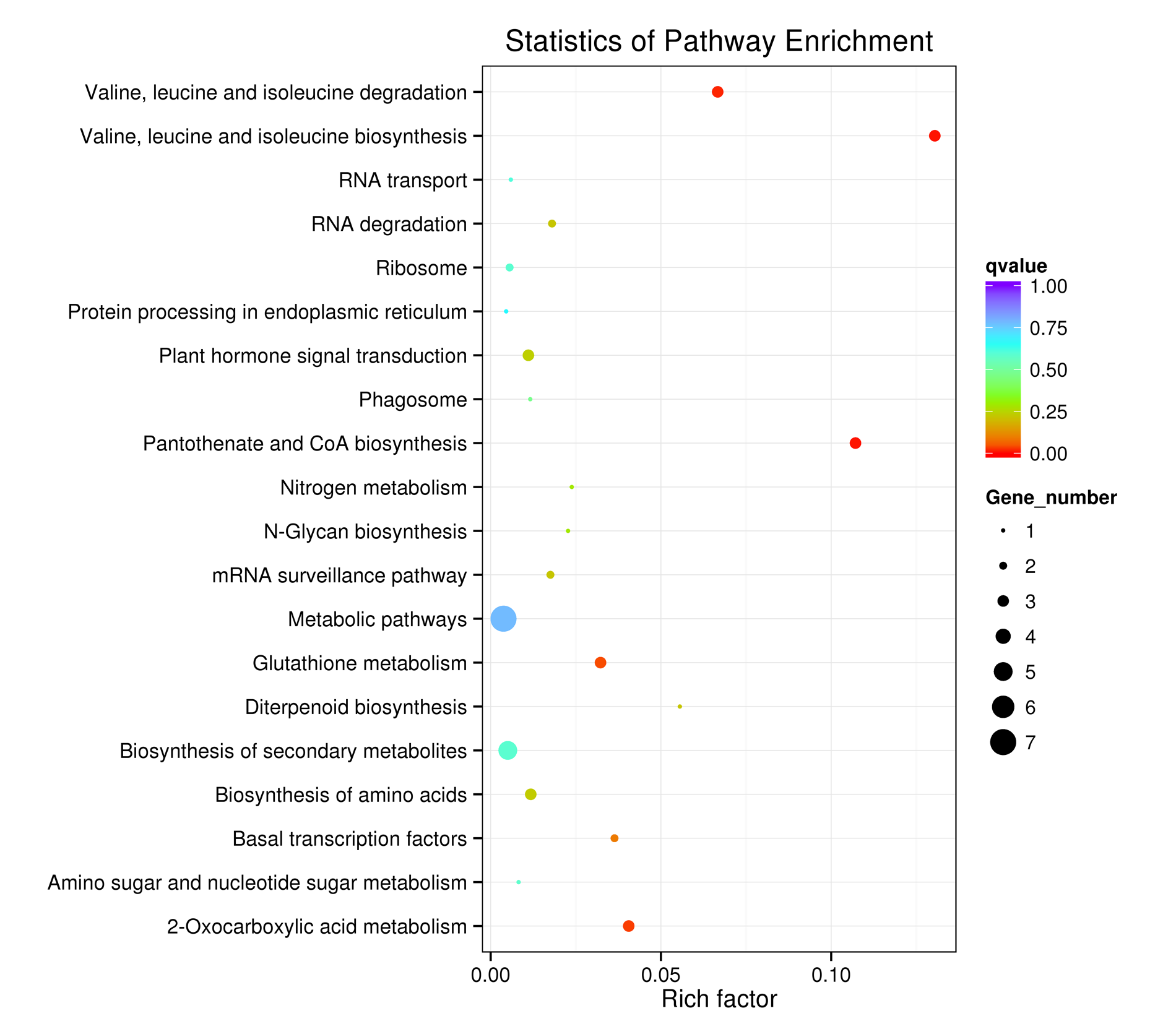

Supplement: Supplementary file 4 — Additional file 4: Figure S4. The top 20 enriched pathways of genes in the associated regions. [file 12864_2020_6558_MOESM4_ESM.tif]
